# Laparoscopic Cholecystectomy in Pregnancy: A Seven-Year Retrospective Study From an Australian Tertiary Center

**DOI:** 10.7759/cureus.50034

**Published:** 2023-12-06

**Authors:** Xinyi Nan, Erick Chan, Kok Sum (Chloe) Wong, Justin Ng, Sara Izwan, Michelle Cooper, Ramesh Damodaran

**Affiliations:** 1 General Surgery, Gold Coast Hospital and Health Service, Gold Coast, AUS; 2 School of Medicine and Dentistry, Griffith University, Gold Coast, AUS; 3 Faculty of Health Sciences and Medicine, Bond University, Gold Coast, AUS

**Keywords:** multidisciplinary care, perioperative care, abdominal shielding, pregnancy, laparoscopic cholecystectomy

## Abstract

Background

Cholecystectomy is the second most common non-obstetric indication for surgery during pregnancy; however, there is little recent literature specifically exploring perioperative care approaches, and a paucity of Australian data exists. This study investigates the incidence of laparoscopic cholecystectomy (LC) during pregnancy, peri-operative management, and post-operative outcomes in a single Australian tertiary center.

Methods

A retrospective analysis of LCs performed on pregnant patients between the ages of 16 and 50 years at a tertiary hospital between 2016 and 2023 was completed.

Results

Twenty-three patients underwent LC. The median gestational age was 17^+4^ weeks (4^+3^-30^+6^). Cases were performed in all three trimesters, with the majority in the second trimester (n=12, 52.2%). Surgery indications were recurrent biliary colic (n=11, 47.8%), acute cholecystitis (n=8, 34.8%), and gallstone pancreatitis (n=4, 17.4%). Obstetrics and Gynecology (O&G) consultations occurred in 56.5% (n=13) of cases. Fetal heart rate (FHR) was recorded perioperatively in 82.6% (n=19) of cases. Preoperative steroids were given to 40% of eligible patients. An intraoperative cholangiogram was performed in 12 (52.2%) cases, of which eight (66.7%) utilized abdominal shielding. There was no perioperative maternal mortality nor fetal loss. Surgical morbidities were pancreatitis (n=1), bile leak (n=1), and intraoperatively recognized bile duct injury (n=1). Two threatened preterm labors and five (26.3%) preterm deliveries occurred.

Conclusion

Performing LC in pregnancy does carry a risk of major morbidity; however, there was no mortality or fetal loss across all trimesters. The decision to perform abdominal shielding during an intraoperative cholangiogram should be approached sensitively in a case-by-case manner, given recent paradigm shifts in radiology. A multidisciplinary approach with standardized local perioperative care policies regarding procedures such as O&G consultation, perioperative steroid use, and FHR monitoring is strongly recommended.

## Introduction

Hormonal changes during pregnancy promote cholelithiasis formation; an increase in cholesterol secretion, a reduction in bile acid secretion, and delayed gallbladder emptying lead to stasis and supersaturation [1]. Gallstones are reported in 5%-12% of pregnant women, and symptomatic biliary disease ranges from 0.05 to 3% [2]. Cholecystectomy is the second most common non-obstetric indication for surgery during pregnancy [3], with an estimated incidence of 1.6% [2]. The maternal surgical complication rate is cited at around 4% across all trimesters, and the fetal rate is at 5.8%, accounting for fetal loss, preterm delivery (PTD), and threatened labor [4,5].

Growing evidence suggests surgical management, compared to conservative management, has reduced maternal and fetal complications for gallstone disease in pregnancy [4,6,7]. A laparoscopic approach is now generally preferred due to the associated reduced hospital stay, financial burden, postoperative maternal morbidity and mortality, and fewer fetal complications; conversion to open is reserved for challenging cases [4,5,8,9].

Traditionally, it has been common practice to delay surgery until the second trimester or the postpartum period; however, there is emerging evidence to support positive outcomes outside this trimester [8]. The Society of American Gastrointestinal Endoscopic Surgeons (SAGES) guidelines recommend laparoscopic cholecystectomy (LC) can be safely performed in pregnant patients during any trimester if indicated [10]. However, definitive guidelines regarding the management of gallstone disease in pregnancy, especially perioperatively, are not strongly evidence-based due to a lack of randomized controlled trials. International guidelines recommend antenatal corticosteroids be given to all women at risk of iatrogenic or spontaneous preterm birth between 24+0 and 34+6 weeks of gestation, including those undergoing non-obstetric procedures given the potential for PTD [11,12].

In Australia, a single 2015 retrospective single-center study investigated outcomes following LC during pregnancy [13], identifying 22 patients over a 10-year period (2003-2013). This number appears to align with international single-center studies from a similar period [5]. Another Australian state population-based study described outcomes of gallstone disease in pregnancy between 2001 and 2012, identifying 239 (12.7%) antepartum cholecystectomies and finding that surgical management was associated with a decreased risk of readmission [14]. There is otherwise a lack of recent data to complement these findings and further demonstrate the incidence and trend of surgical management in pregnancy in the Australian cohort. Furthermore, overall, there appears to be a paucity of literature exploring perioperative care approaches such as consultation with Obstetrics and Gynecology (O&G), fetal heart rate (FHR) monitoring, and steroid use pre-operatively.

## Materials and methods

A retrospective observational analysis of all emergency and elective laparoscopic cholecystectomies performed on pregnant patients between the ages of 16 and 50 at a public tertiary teaching hospital between January 1, 2016, and January 1, 2023, was performed. Open cholecystectomies were excluded. Patient demographics and operative data were obtained from the data analytics team. Pre-operative management and post-operative outcomes were collected from the electronic medical record.

Patient demographic data collected included age, body mass index, smoking and diabetic status, and trimester of pregnancy at the time of operation. Operative data include indication for surgery (acute cholecystitis, chronic cholecystitis, biliary colic, gallstone pancreatitis), length of surgery, time from decision for theatre to operation, whether an abdominal shield was used, whether an intraoperative cholangiogram (IOC) was performed, the presence of choledocholithiasis, and intraoperative spillage. Pre-operative management was reviewed for consultations from the O&G team and/or midwives, use of pre-operative steroids, a record of cardiotocography, or FHR monitoring peri-operatively. Post-operative outcomes, including surgical and obstetric complications, neonatal complications, and birthing outcomes, were collected.

Local ethics approval was obtained through the local Human Research Ethics Committee. HREC Reference: HREC/2023/QGC/90845 and SSA Reference: SSA/2023/QGC/90845. All de-identified data was securely stored on a password-encrypted drive.

## Results

Twenty-three pregnant patients underwent LC between January 1, 2016, and January 1, 2023, with none requiring conversion to open. The median maternal age was 27 years (19-41), and the median estimated gestational age at the time of surgery was 17+4 weeks (4+3-30+6). Twelve patients (52.2%) had surgery performed within the second trimester, six (26.1%) in the first trimester, and five (21.7%) in the third trimester.

**Figure 1:**
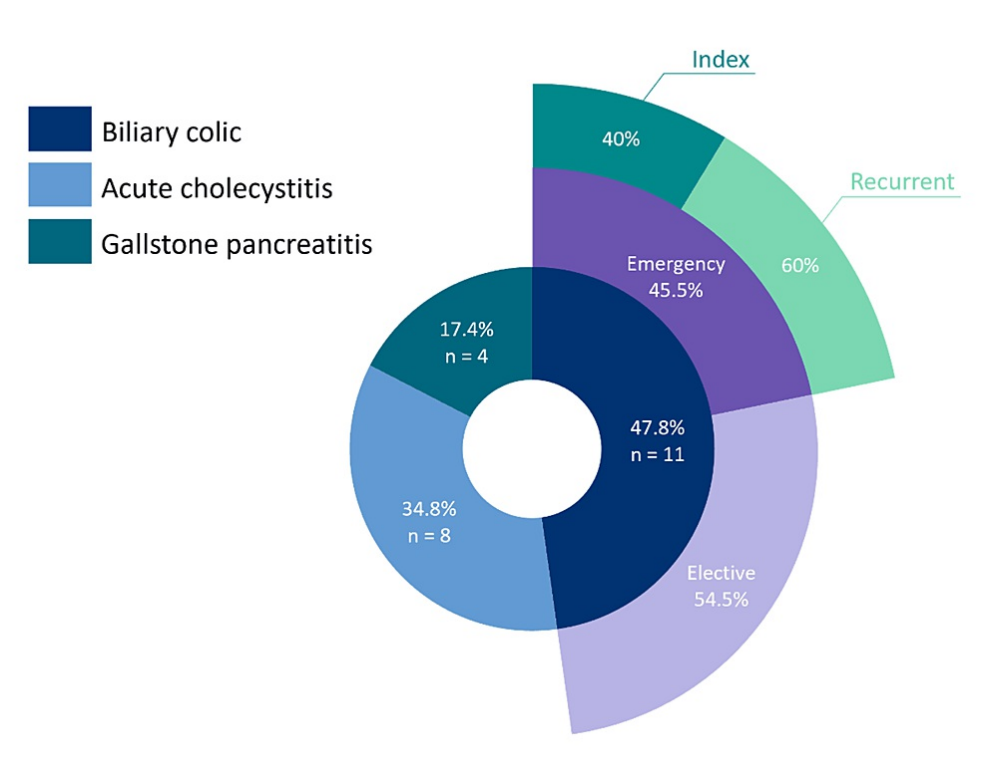
Indications for surgery: biliary colic, acute cholecystitis, and gallstone pancreatitis. For biliary colic, whether surgery was performed in an emergency (n=5) or elective (n=6) setting and whether emergency surgery was performed at an index admission (n=2) or after recurrent presentations (n=3)

The most common indication for surgery is biliary colic, followed by acute cholecystitis and gallstone pancreatitis (Figure 1). Eleven patients (47.8%) underwent surgery for severe biliary colic, of which six underwent elective surgery for recurrent biliary colic; five emergency LCs were performed, with three for recurrent presentations and two at index admission. Five (62.5%) patients with acute cholecystitis underwent LC on index admission; two had representations. One patient underwent a semi-elective procedure four days after presentation. Of the four patients with gallstone pancreatitis, three had recurrent acute presentations before the emergency operation. One patient was conservatively managed for a first-episode attack and underwent elective LC during the second trimester.

There were 15 cases (65.2%) of emergency LC and eight (34.8%) elective LC; all elective cases were performed in the second trimester. The median time from decision to operate to operation for emergency cases was 1 day (0-3), and for elective cases, it was 19.5 days (4-42). The median duration of operating time was 83 minutes (47-203). Twelve cases (52.2%) had IOC performed, with eight (66.7%) having documented use of an abdominal shield and six (50%) having choledocholithiasis found on the IOC. One underwent endoscopic retrograde cholangiopancreatography (ERCP) after an unsuccessful attempt at stone retrieval via guidewire and catheter. Three cases of choledocholithiasis were successfully flushed intraoperatively. One case had intraoperative transcystic common bile duct (CBD) exploration performed with successful flushing of choledocholithiasis. One was referred for ERCP for a 3mm distal CBD stone but was conservatively managed due to the small stone size. No IOC was performed in the third trimester. Radiation exposure in milligray (mGy) and screen time data from IOC were available for seven (58.3%) cases, with an average exposure of 2.4 mGy and a screen time of 11 seconds. 

**Figure 2:**
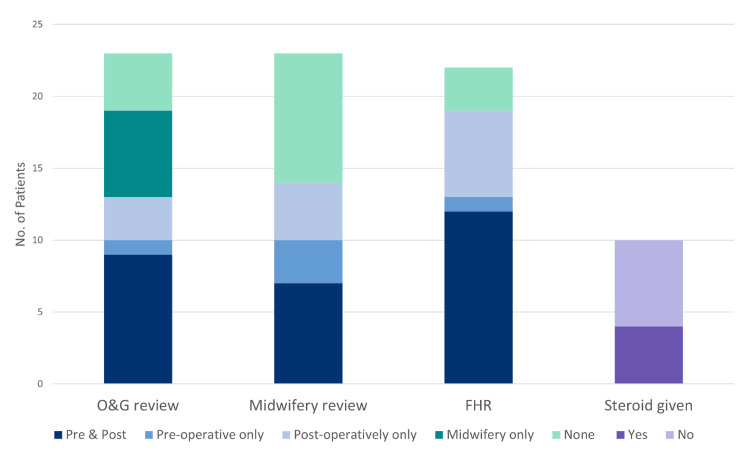
Perioperative management of patients in terms of review of obstetrics and gynecology (O&G) and/or the midwifery team, recording of fetal heart rate (FHR), and whether steroid was given in those with gestation 24+0 to 34+6 weeks

Consultations from O&G were sought for 13 (56.5%) cases (Figure 2). Three (13.6%) of the 22 patients with gestation greater than or equal to 7+0 did not have FHR recorded perioperatively. Four (40%) of ten patients with gestations from 24+0 to 34+6 were given preoperative steroids (two doses of betamethasone 11.4 mg intramuscularly 12-24 hours apart).

The median length of stay was three days (1-24; interquartile range = 5). There were no perioperative maternal or fetal mortalities. Surgical post-operative complications occurred in three (13%) cases. There was one bile leak that required a return to the theater with a transcystic biliary stent, suture repair, and ERCP. The other complication was a common hepatic duct injury identified intraoperatively during transcystic exploration. The patient underwent ERCP the next day for stone retrieval with no leak identified and an uneventful recovery post. One patient experienced pancreatitis, requiring ERCP and stent insertion. There were two cases (8.7%) with obstetric postoperative complications in the form of threatened preterm labor (TPTL) at gestation of 30+2 and 24+6, requiring steroids and nifedipine postoperatively for one at day zero and the other at day one.

Birth records were not available for three patients (13.0%) due to a lack of follow-up. One had a termination of pregnancy as per personal choice at eight weeks of gestation. Of the remaining 19 births, the median gestation at delivery was 38+5 weeks (34+0 to 40+4). There were five (26.3%) preterm deliveries with no documented neonatal complications. There were 13 (68.4%) spontaneous vaginal deliveries. Three (15.7%) underwent elective caesarean sections (CS), of which two were secondary to intrauterine growth restriction. Three (15.7%) underwent emergency CS, one of which had preterm prelabour rupture of membranes; the other two failed to progress. These emergency CS occurred at 141, 103, and 98 days, respectively, after undergoing LC. 

## Discussion

There is an inherent reluctance to perform non-obstetric surgeries during pregnancy. A recent American nationwide retrospective cohort study has revealed that contrary to current national and international guidelines favoring surgical management, most pregnant women admitted in the United States with acute cholecystitis are managed nonoperatively [7]. 

Weighing up the risks and benefits for both mother and fetus often poses a management dilemma. Biliary colic could be managed conservatively in a pregnant patient. However, this presents a risk of recurrent hospital admissions, progression to complicated gallstone disease, and an impact on quality of life [15,16]. The risks of acute cholecystitis in pregnancy include miscarriage, preterm delivery, and stillbirth from peritonitis. Other risks include gallstone pancreatitis and cholangitis, which require timely surgical treatment [8]. 

In this study, a total of 23 patients underwent LC over a seven-year period, equating to an average of 3.2 cases/year, which is comparable to existing literature on single tertiary center studies [13,17-19]. There was neither maternal mortality nor recorded fetal loss. There was one minor morbidity of postoperative pancreatitis and two major surgical morbidities of bile duct injury, with one requiring take-back to the theater. There was no direct effect on long-term pregnancy outcomes. 

A conversation about the open cholecystectomy rate of 2.2% has been cited in the literature [5]. However, in this study, there was no conversion to open laparoscopy. 

The SAGES guideline recommends protective shielding should be used in every case with IOC to optimize shielding of the fetus, although no specific complications have been identified in the fetus with radiation exposure of less than 50 mGy; this is much higher than the standard IOC and ERCP exposure dose of 0.1 mGy [10, 20]. Lead shields should be placed underneath the patient’s abdomen, as the X-ray beam originates from beneath the patient. The practice of a second shield placed over the abdomen is unproven [21]. The experience of the radiographer is important in minimizing screen time and optimizing the positioning of the X-ray with angulation through the shields towards the biliary tree.

Interestingly, however, there has been a recent paradigm shift away from routine contact shielding in radiology, which has been echoed by positional statements from several international radiology professional societies [22-25]. It is thought that fetal contact shielding has limited value for fetal protection as most of the fetal/embryo exposure results from internal scatter in the tissues of the mother, and shields may compromise the diagnostic efficacy of the exam, resulting in an actual increase in the radiation dose required [22,25]. It is, however, important to consider that most of these cases refer to exposure to radiation at the time of general diagnostic radiography and do not specifically refer to the use of IOC in pregnancy or where the shield is placed outside of the field of view. In our study, the average radiation exposure is low (2.4 mGy), and the screening time is short (11 seconds). However, only one case has detailed the correct positioning of the shield beneath the patient’s lower abdomen, while the rest only documented that shielding was used. Given the recent shift in guidelines, discussions regarding utilizing shields should be handled sensitively and may be at the discretion of the operating surgeon.

Twelve cases (52.2%) in the current study were performed in the second trimester, which is traditionally considered the optimal time for laparoscopic intervention in pregnancy [12]. The first (n=6) and third trimester (n=5) data add to the growing literature that supports the safety of laparoscopic intervention in any trimester and aligns with current international guidelines [10]. Of note, a few recent population-based cohort studies have cited increased risk of PTD in patients undergoing LC in the third trimester, with one identifying associated increased maternal and fetal complications compared to other trimesters [26], another compared with postpartum operations [27], but a third did not identify any significant difference in complications [28].

In this study, two PTDs (both at 36+6) occurred at six weeks or more post-LC in the third trimester and were planned induction of labors due to obstetric concerns (preeclampsia and intrauterine growth restriction). Three PTDs (34+0, 36+0, 36+3) occurred in those operated on during the second trimester, with all three cases having had certain high maternal risk factors for preterm delivery (personal history of preterm labor, history of intrauterine fetal demise, and poorly controlled type-1 diabetes). There were no other recorded neonatal or maternal complications. It is difficult to ascertain whether these are directly connected to surgical intervention, with all the PTDs occurring more than 30 days after surgery. Nonetheless, the sample size in our study is small, and greater numbers are required for further analysis and longer-term follow-up for neonatal outcomes.

A 2022 Swedish survey to address patients’ perceptions and experiences of intervention for gallstone disease during pregnancy revealed a strong sentiment that inadequate information was given about their condition and upcoming surgery [29]. Decision-making in the understandably delicate situation of non-obstetric surgery in pregnancy should involve a multidisciplinary team, including pre-anesthetic risk discussion with the surgeon, anesthetist, and O&G to help the patient make informed decisions. Neonatologists should also be notified in selected cases should preterm delivery occur perioperatively. 

Not every case in our study had consultations from O&G; four cases did not have any documented input, while six had midwifery reviews only. Preoperative and postoperative FHR monitoring, considered the standard for a viable fetus (>22 weeks), was achieved in only 70% of cases, with the rest having documented postoperative FHR only. 

Given the potential risk of PTD with non-obstetric surgery, current international consensus suggests prophylactic corticosteroids be given to those between 24+0 and 34+6 weeks of gestation [11,12]. Of the six patients within that gestational timeframe who did not receive preoperative steroids, four did not have any pre-operative O&G review. It is reassuring that there were no cases of immediate PTD associated with surgery. However, two cases of TPTL did occur, one on day zero and another on day one postoperatively; both resolved with steroids and tocolysis. There also needs to be caution regarding offering preoperative steroids in patients with systemic infections, given their effect on the maternal immune system, and this should be a discussion between the O&G, the surgeon, and the patient, which further supports a multidisciplinary approach.

Most data in the current literature are found in case series, retrospective studies, and meta-analyses, leading to varying management approaches in part dictated by the surgeon and the patient’s personal preferences. Limitations of this study include the retrospective nature and reliance on appropriate electronic coding systems to capture patient data. The small sample size is another limitation, and it may not capture those who were successfully conservatively managed or referred for surgery elsewhere. The small size also inflates the bile leak rate (n=1, 4.3%); it is recognized that the overall leak rate for LCs performed for all patients across the same study period was not available for this study, thus limiting the ability to make comparisons. It may be of benefit for future studies to explore longitudinally preterm neonatal outcomes in association with cholecystectomy in pregnancy and capture a wider population through state-based or Australian nationwide cohort analyses. 

## Conclusions

This study not only complements international literature in demonstrating that LC is safe in pregnancy but also further demonstrates that a multidisciplinary team approach is required in perioperative optimization. Each health service requires formal local policies regarding O&G consultation, perioperative monitoring, and steroid use. Intraoperative measures such as abdominal shielding if a cholangiogram is performed could now be a contentious point given recent paradigm shifts in radiology and should be approached sensitively in a case-by-case manner. This adds to the current limited Australian literature supporting the trend of safe and positive outcomes for LC in pregnancy.
